# Multiple sites of adaptation lead to contrast encoding in the *Drosophila* olfactory system

**DOI:** 10.14814/phy2.12762

**Published:** 2016-04-06

**Authors:** Jon Cafaro

**Affiliations:** ^1^ Department of NeurobiologyDuke UniversityDurhamNorth Carolina; ^2^ Department of BiologyDuke UniversityDurhamNorth Carolina

**Keywords:** Adaptation, *Drosophila*, Olfaction, Weber's law

## Abstract

Animals often encounter large increases in odor intensity that can persist for many seconds. These increases in the background odor are often accompanied by increases in the variance of the odor stimulus. Previous studies have shown that a persistent odor stimulus (odor background) results in a decrease in the response to brief odor pulses in the olfactory receptor neurons (ORNs). However, the contribution of adapting mechanisms beyond the ORNs is not clear. Thus, it is unclear how adaptive mechanisms are distributed within the olfactory circuit and what impact downstream adaptation may have on the encoding of odor stimuli. In this study, adaptation to the same odor stimulus is examined at multiple levels in the well studied and accessible *Drosophila* olfactory system. The responses of the ORNs are compared to the responses of the second order, projection neurons (PNs), directly connected to them. Adaptation in PN spike rate was found to be much greater than adaptation in the ORN spike rate. This greater adaptation allows PNs to encode odor contrast (ratio of pulse intensity to background intensity) with little ambiguity. Moreover, distinct neural mechanisms contribute to different aspects of adaptation; adaptation to the background odor is dominated by adaptation in spike generation in both ORNs and PNs, while adaptation to the odor pulse is dominated by changes within olfactory transduction and the glomerulus. These observations suggest that the olfactory system adapts at multiple sites to better match its response gain to stimulus statistics.

## Introduction

Persistent increases in sensory stimuli, like the increase in light level as the sun rises, are common in nature. Such increases in the mean (background) level of the stimulus are often accompanied by increases in variance of the stimulus. To adapt to changes in both the mean and variance of the stimulus, sensory systems often decrease their responsiveness to both the persistent background and additional transient increases in the stimulus (for review, see Wark et al. 2007; Rieke and Rudd 2009; Carandini and Heeger 2012). This adaptation matches the system's response range to the changing stimulus statistics (Barlow 1961; Laughlin^1981^). Changes in the background level of olfactory stimuli are common in nature and accompanied by increases in the variance of odor stimuli (Murlis et al. 2000). Studies in olfactory receptor neurons (ORNs) have examined how odor background alters responses to further odor stimulation (Reisert and Matthews 1999; Martelli et al. 2013; also see: Kurahashi and Menini 1997; Dolzer et al. 2003; Nagel and Wilson 2011; Murmu et al. 2011; Burgstaller and Tichy 2012); but studies in neurons downstream of the ORNs have focused on other odor stimuli (Wilson 1998; Brown et al. 2005; Kadohisa and Wilson 2006; Wilson et al. 2006; Bhandawat et al. 2007; Lecoq et al. 2009; Das et al. 2011; Saha et al. 2013). Thus, it is unclear how later stages in the olfactory circuit adapt to transient odor increases during a background odor and how this adaptation compares with that in the ORNs. Understanding how adaptive mechanisms are distributed within the early olfactory circuit provides insight into how neural computations are distributed to handle sensory challenges.

Two methods are common for probing adaptation. First, is to examine how the neural response decreases during the background stimulus (herein referred to as “background adaptation”). Second, is to examine how the neuron reduces its response to rapid stimulus increases delivered during the background stimulus (herein referred to as “pulse adaptation”). Some studies have shown that these two processes can occur independently while others suggest a connection (for review, see Wark et al. 2007). Moreover, pulse adaptation in the visual system requires multiple sites to handle the challenges posed by changes in the background stimulus (Laughlin et al. 1987; Dunn et al. 2007). These considerations suggest that independent mechanisms at multiple levels of the olfactory neural circuit might be required to encode changes in natural odor statistics.

I used the well‐understood *Drosophila* antennal lobe circuit to study adaptation in the ORNs and the second‐order neurons (projection neurons, PNs). In this system, I measured adaptation in four stages: (1) in the ORN local field potential (LFP), (2) ORN spikes, (3) PN synaptic potential, and (4) PN spikes. Comparing the magnitude of both the background and pulse adaptation at these four stages under identical stimulus conditions allowed me to identify adaptation in distinct neural processes: (1) olfactory transduction, (2) ORN spike generation, (3) the glomerular transform, and (4) PN spike generation (Fig. 2A, B top). I find that background adaptation is primarily mediated by changes in spike generation in both ORNs and PNs. However, pulse adaptation is primarily mediated by changes in olfactory transduction and the glomerular transform. PNs adapt more than ORNs to both background and pulse stimuli. Background and pulse adaptation combine to enable contrast encoding in the PN spike rate. This study illustrates the importance of distributed adaptive mechanisms in neural computation and shows that the olfactory system, like the visual system, can faithfully encode stimulus contrast.

## Materials and Methods

### Fly strains and neural recordings

All fly strains and recording techniques used in this study have been previously described and are briefly described below.

ORN activity was recorded, as described earlier, using single‐sensillum recordings (Kaissling 1995; de Bruyne et al. 1999; Bhandawat et al. 2007). Briefly, a sharp electrode filled with an extracellular saline solution (in mM: 103 NaCl, 5 KCl, 5 Tris acid, 10 Glucose, 26 NaHCO_3_, 1 NaH_2_PO_4_, 1.5 CaCl_2_, 4 MgCl) was inserted into a sensillum to perform extracellular recordings. ORNs projecting to VM7 glomerulus are present in the pb1 (palp basiconic 1) sensilla along with another ORN, which expresses the Or71a receptor. I performed ORN recordings in the Or71a deletion mutant (Shiraiwa et al. 2002) to eliminate spikes from the Or71a‐ORN and the need for spike sorting. In all but four of the 40 recordings, I only observed one spike waveform (data not shown). In the remaining four ORNs, the second spike waveform occurred at a very low rate (~2 Hz) and was not modulated by odors; thus, the error due to counting Or71a spikes as VM7‐ORN spikes was small.

For PN recordings, I targeted VM7 PNs in the antennal lobe of NP3481‐GAL4:UAS‐CD8GFP flies in which a subset of PNs, including VM7, express GFP. Recordings were targeted to GFP‐positive PNs and VM7s were identified based on a strong response to low‐intensity pulses of 2‐butanone. PN activity was recorded in a current‐clamp mode using whole‐cell patch‐clamp technique using patch electrodes (resistance ~10 MΩ; electrodes filled with (in mM): 140 K‐aspartate, 1 KCl, 10 HEPES, 1 EGTA, 0.5 Na_3_GTP, 4 MgATP). An extracellular saline solution (the same as the one used in the ORN sharp recordings) was bubbled with 95% O_2_/5% CO_2_ and perfused onto the brain during PN recordings. PNs were recorded with resting potentials approximately between −40 and −30 mV. Spikes in VM7 PNs were generally small (~1–10 mV) but they had distinct temporal profiles that allowed automated identification. Both ORN and PN recordings were acquired with an A‐M Systems Model 2400 Amplifier with a 100 MΩ head stage. Data was sampled at 10 kHz using a National Instruments analog‐to‐digital converter (NIDAQ PCIe‐6351).

### Olfactory stimuli

**Figure 1:**
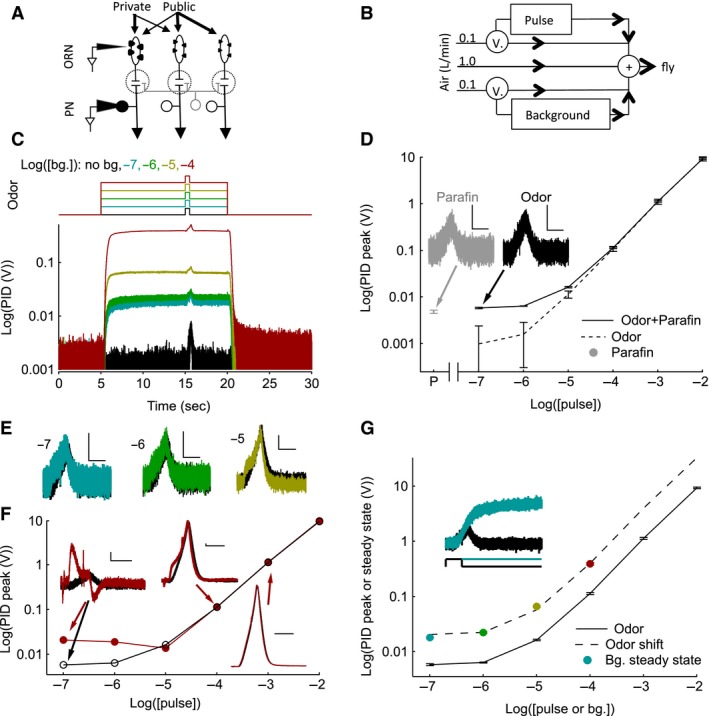
Odor stimulation and measurement of stimulus using photoionization detector (PID)**.** (A) Private odor predominately activates ORNs presynaptic to VM7 PNs and thus minimizes lateral interactions; and a public odor activates multiple ORN classes. (B) Schematic illustrating the air flow pathway and valves (V.) used to control the odor stimuli. (C) The average (*N* = 10 trials) PID responses (bottom trace) to 2‐butanone odor stimulus protocols used (top trace) to measure neural activity. Each trace shows the response to background and pulse odor at the same concentration (10^−7^ pulse on 10^−7^ background etc.). D‐F, Quantification of the pulse stimulus. The response of the PID to background odor is subtracted. (D) The average pulse response of the PID without (solid) or with the paraffin oil response subtracted (dashed lines) is plotted as a function of pulse odor concentration. At low odor concentrations, the PID response is dominated by the solvent, paraffin oil. Within margins of error, the PID responses are linearly related to the odor concentration. “P” on the *X*‐axis is paraffin oil alone. Error bars indicate Standard error of the means (SEMs). Gray point, indicates the PID response to the paraffin oil pulse alone. Inset, shows that the paraffin oil PID response (gray) is similar to the response to 2‐butanone at 10^−7^ concentration, which includes responses due to both paraffin oil and odor (black). Scale bars are 5 mV, 500 msec. (E) The PID pulse response as a function of time in the presence of background odor (colored) and its absence (black) shows that PID responses are similar with and without background. Background and pulse odor intensity are the same and listed to the left of each trace. Scale bars are 5 mV, 500 msec. F, The average peak PID pulse response during the highest background odor stimulus (10^−4^; red points and line) and in its absence (black circles). Insets show the PID pulse responses as a function of time in the presence (red) and absence (black) of the odor background for a select set of responses. Scale bars are 5 mV, 500 msec. G, The average peak PID pulse response in the absence of a background stimulus (black points and solid curve) and the steady‐state PID voltage during the background of the same odor concentration. The dashed line is a shifted version of the PID pulse peak curve obtained by multiplying a single scale factor (~4) chosen to match the PID steady‐state response. The pulse and background concentrations are, therefore, related to each other by a single scale factor. The inset shows the PID pulse response (black) at 10^−7^ concentration and the PID response during the background stimulus of the same intensity (blue).

Olfactory stimuli used during ORN and PN recordings were identical. All experiments except those in Figure 8 utilized 2‐butanone (Sigma‐Aldrich) as the odor stimulus. 2‐butanone was chosen because at most odor intensities used in this study, it strongly activates pb1A and minimally activates other ORN classes (Olsen et al. 2010). The odor used in Figure 8 is ethyl acetate (Sigma‐Aldrich), which was chosen because, at the odor intensities used in this study, it activates pb1A but also strongly activates many other ORN classes (Hallem and Carlson 2006; Olsen et al. 2010). Here, I term 2‐butanone a “private odor”, because the dominant circuit activated is a single class of ORN providing input to a single glomerulus (Fig. 1A). Alternatively, I term ethyl acetate a “public odor”, because it activates more ORN classes at the intensities used (Olsen et al. 2010).

Odors were diluted in paraffin oil (J.T. Baker) and prepared both as pulse vials (5 mL in 20 mL glass scintillation vials) and background bottles (200 mL in 250 mL glass VWR bottles). Large background bottles were necessary to maintain constant background odor intensity. Three‐way solenoid valves were used to switch air pathways while maintaining a constant rate of airflow, regardless of changes in the odor stimulus (Fig. 1B; 1L per min. clean air stream +100 mL per min. background air stream +100 mL per min. pulse air stream).

Stimulus trials were performed in repeated trial blocks (6–46 trials) using a selected combination of one pulse (10^−7^,10^−6^,10^−5^,10^−4^,10^−3^,10^−2^) and one background (10^−7^,10^−6^,10^−5^, 10^−4^) odor intensity. Each trial was 30 sec long with 1 min. in between trials to allow the headspace to refill. A trial consisted of 5 sec prestimulus, 15 sec background, 500 msec pulse, and 10 sec poststimulus periods (Fig. 1C top). On alternate trials, the pulse was delivered without the background stimulus and data were only used if sequential trials with and without background were obtained. Because recordings lasted for a limited time (<4 h), only odor responses to 1–5 pulse odor intensities at a single background odor intensity could be measured during a single recording.

### Photoionization detector measurement

A photoionization detector (PID; Aurora Scientific Inc. miniPID model 200A) was used to measure the odor stimulus. During recording, background and pulse stimuli were controlled independently while maintaining a constant rate of airflow (Fig. 1B). For PID measurements, I kept the recording conditions similar to those used in neural recordings. The PID was placed at the same spot as the fly and the PID vacuum pump rate was set similar to that of the flow meters (“high”).

To assess the temporal dynamics of the background and pulse odor (Fig. 1C), the response of a PID was measured to background and pulse odor at the same intensity. Similar to other studies (Nagel and Wilson 2011; Martelli et al. 2013), a delay in the onset of the PID response and a slowing of the response kinetics compared to the valve command was observed. Importantly, the PID response remained stable and did not decay during the background stimulus (Fig. 1C).

Next, the linearity of the pulse odor concentration was examined (Fig. 1D). The PID response was a sum of the individual responses to the odor and to the solvent, paraffin oil (Fig. 1D solid line). The paraffin oil response dominated the overall response at low odor concentrations (Fig. 1D inset). After subtracting the paraffin oil response, the concentration–response relationship of the PID was linear (within error bars) across the entire range of odor concentrations (Fig. 1D dashed line).

I then examined if the addition of the background stimulus changed the strength of the pulse response (Fig. 1E, F). First, I compared the effect of background odors at the same concentration as the pulse for all background concentrations used in this study, that is, the effect of background at 100% contrast. I observe very similar PID responses in the presence and absence of background at 100% contrast (Fig. 1E). Second, I compared the PID response to all six pulse concentrations delivered during the highest background concentration (10^−4^) to the corresponding response without background odor. I used these data to assess the worst‐case scenario. I found that the pulse response at 10^−5^ and higher intensities are unaffected at 10^−4^ background. However, at 10^−6^ and 10^−7^ odor intensities, there is a large change in the PID kinetics and amplitude (Fig. 1F). This is likely due to a contamination of the pulse tubing by the background odor where both independent lines connect to the final air tube (Fig. 1B). Overall, the PID responses with and without background are very similar except when the background is >first‐order of magnitude higher than the pulse (i.e., pulse stimuli ≤1% contrast). Odor contrast ≤1% occurs in only 3/24 of the pulse‐background stimuli tested, and in these cases, the unintended increase in the stimulus proved to be inconsequential to the cells' neural responses, which remained near zero (i.e., decreasing the pulse odor intensity to correct for the contamination would be very unlikely to change the measured results).

Finally, I compared the PID response elicited by the background to that of the pulse response (Fig. 1G). Comparing the same background and pulse odor concentrations, the steady‐state background responses were significantly larger than the peak pulse PID measures. This difference is likely because the pulse time is too short to allow the odor concentration to reach its maximum (Fig. 1G inset). Differences between the background and pulse concentrations suggests an error in the calculation of contrast (pulse concentration/background concentration) in Figure 6,7 and calls into question, the validity of comparisons between pulse response curves at different backgrounds on a contrast scale. However, the steady‐state background PID measures were consistently at the same distance on a log scale from the pulse peak value of the same concentration (the background steady states are ~4 times larger than the pulse peaks). In fact, the steady‐state values were well fit by a shifted version of the pulse PID response curve (Fig. 1G dashed line). This indicates that although the precise values of contrast calculated by concentration (e.g., the *X*‐axis of Figure 6, 7) are slightly off, the comparison between curves is valid because the actual and nominal concentrations are related by a scaling factor. Thus, the observations made are a result of biologic factors and not of misestimating the odor stimulus.

### Data analysis

**Figure 2:**
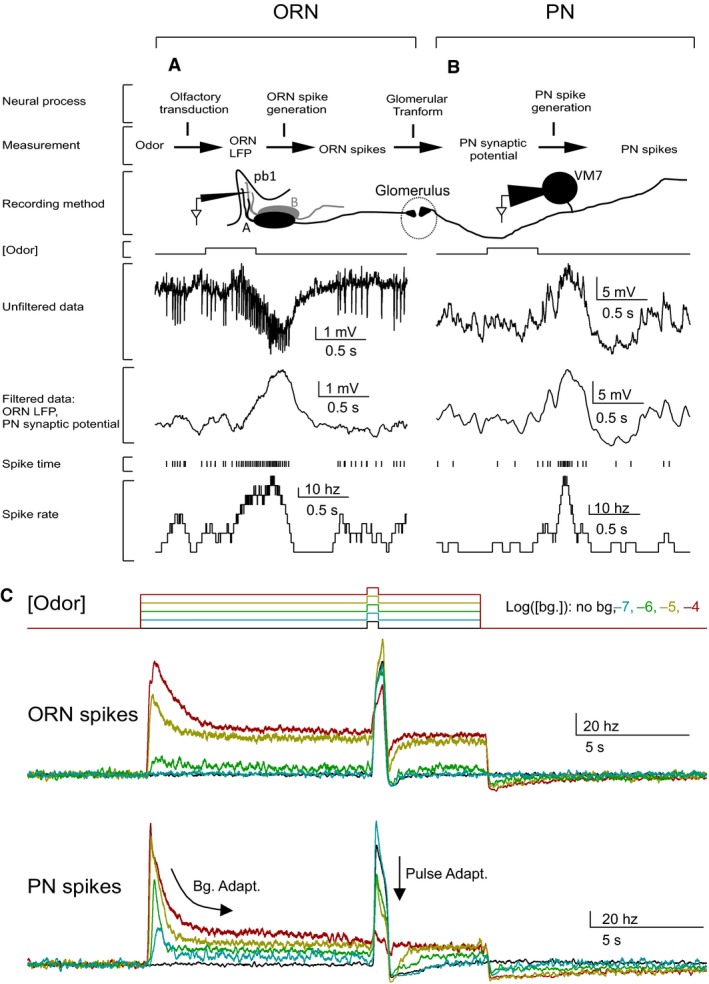
Experimental design and basic phenomena. A‐B. Adaptation is measured at four levels in the circuits. Two of the signals are obtained from ORN recordings and are shown in panel A. The other two signals are obtained from PN recordings and shown in panel B. (A) ORN responses were recorded with a sharp electrode inserted into the pb1 sensilla. pb1 sensilla in the Or71a mutants have one functional ORN, pb1A, and one nonfunctional, pb1B. Sample trace shows the response of a pb1A ORN to a pulse of 2‐butanone ([10^−6^]). Response to odor consists of a slow signal, as well as, spikes. Smoothing isolated the slow signal, which is a measure of the transduction step. Spikes are measured separately. (B) PN responses were recorded with whole‐cell patch‐clamp and data were analyzed similarly to the ORN. (C) The background odor intensity is color coded and indicated at the top right of the panel. Top traces show a schematic of the odor stimulus command. Bottom traces, show ORN and PN spike responses to a 10^−4^ pulse during a range of background odor intensities. Responses were averaged across trials and cells. ORN spikes are an average of (*N* = 4–11 trials) and (*N* = 5–6 cells). PN spikes are averaged across (*N* = 3–10 trials) and (*N* = 5–7 cells).

To understand olfactory processing at several neural stages, four signals were analyzed: the ORN LFP, ORN spike rate, PN synaptic potential, and PN spike rate before odor onset and during the background and pulse odor stimuli. To examine the ORN LFP or the PN synaptic potential, the raw voltage traces were averaged over a 50 msec sliding window (Fig. 2A, B). This procedure substantially decreases the spike amplitude relative to the low‐frequency voltage changes. Alternatively, to estimate spike rates, spikes were detected using custom spike detection algorithms (written in MATLAB R2011b). Spike rates were reported as averages over a 100 msec sliding window. All four signals showed spontaneous activity before the odor stimulus. To focus on changes resulting from the odor stimulus, the spontaneous activity (average over 5 sec before the odor stimulus began) on each trial was subtracted for all four signals analyzed.

**Figure 3:**
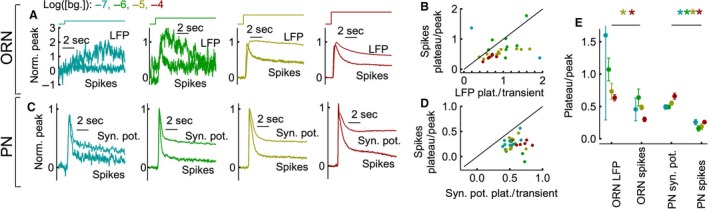
Adaptation of the odor background response. (A) The average (*N* = 5–9 cells, 3–63 trials per cell) normalized ORN LFP and spike rate response at different odor backgrounds. (B) The ratio of plateau to transient peak spike rate plotted against the plateau to peak ratio for the ORN LFP shows that the adaptation is stronger at the level of ORN spikes. Error bars are not shown for clarity. (C‐D), The same as A‐B but for PNs (*N* = 5–9 cells, 4–39 trials per cell). (E) Average plateau/peak ratio at each neural stage and background odor intensity shows greater adaptation in the ORN spikes than ORN LFP at the high background concentration. Background adaptation is strongest at the level of PN spikes. Error bars are Standard error of the means (SEMs) across cells. Asterisks indicate significant differences between paired data in panels B, D at each background odor intensity (*P* < 0.05 in a paired t‐test).

**Figure 4:**
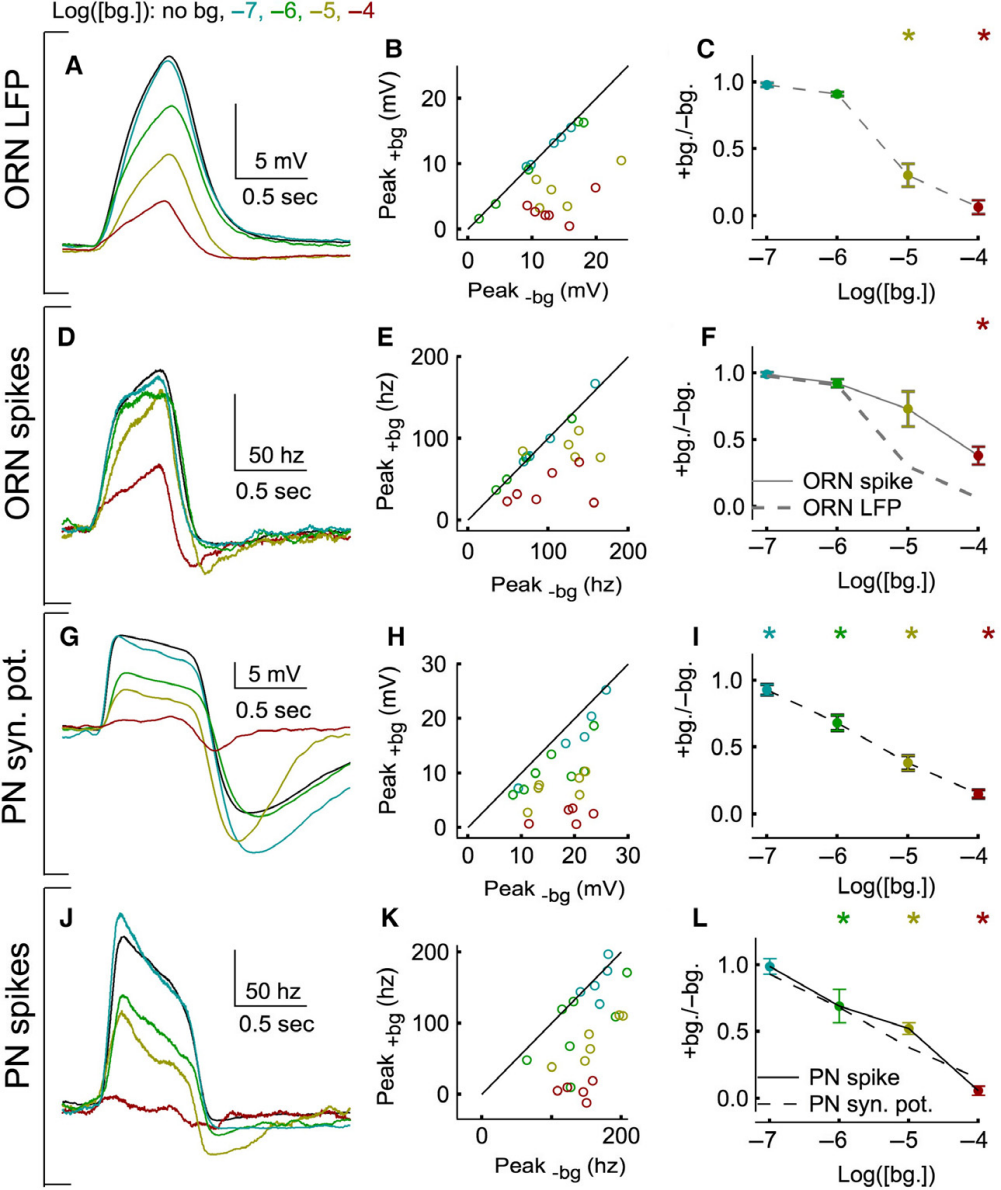
Adaptation of the pulse response at one odor pulse intensity (10^−4^) (A) ORN LFP pulse response averaged across trials (*N* = 4–11 trials) and cells (*N* = 5–6 cells). The pulse response was calculated by subtracting the background activity in the 5 sec preceding the pulse. (B) The peak of the pulse response in each cell in the absence (*X*‐axis) and presence (*Y*‐axis) of the background odor shows that there is significant adaptation only for the two highest concentrations. Error bars are not shown for clarity. (C) The peak of a cell's pulse response in the presence of a background odor divided by its peak in the absence, averaged across all cells. Error bars are Standard error of the means (SEMs). Asterisks indicate significant adaptation between paired data in panel B (*P* < 0.05 in a paired t‐test) for a given background odor intensity. (D‐F) As in A‐C, but using ORN spike rate data (*N* = 4–11 trials and 5–6 cells). Lines show interpolated fits from panels C and F. G‐I, As in A‐C, but using PN synaptic potential data (*N* = 3–10 trials and 5–7 cells). J‐L, As in A‐C, but using PN spike rate data (*N* = 3–10 trials and 5–7 cells). Lines show interpolated fits from panels I and L.

Cells responded to the background odor stimulus with transient changes from rest that decayed and mostly plateaued within 5 sec (see Fig. 2; Fig. 3). Neural responses to the 500 msec pulse odor stimulus were mostly over within 1 sec of the odor pulse onset (see Fig. 2; Fig. 4). To characterize the odor responses, I quantified three parameters on each trial: (1) the peak of the background response, (2) the plateau of the background response, and (3) the peak of the pulse response. The peak of the background and pulse responses were quantified by averaging over a 100 msec window around the mean response maximum, within either 1 sec of the background odor or pulse odor onset. The time of the peak differed between cells, stimulus conditions, and the signal being analyzed, so the peak was estimated independently for each cell, stimulus condition, and signal type. The plateau during the odor background was quantified as the average activity level (calculated over 5 sec) beginning 5 sec after the background stimulus began. To separate the pulse response from the background odor response, the background plateau response was subtracted from the peak of the pulse response on each trial. Standard error of the means (SEM) were calculated for each parameter (eq. (1)):

(1) $$\sigma_{\overset{¯}{x}}=\frac{\sigma_{x}}{\sqrt{N}}$$

where N equals the number of samples of parameter *x* and *σ*_*X*_ equals the unbiased standard deviation of parameter *x*.

The three measured response parameters described above were then used to estimate background and pulse adaptation factors in each stimulus condition and cell. To estimate background adaptation, the plateau was divided by the peak of the background response (Fig. 3). To estimate pulse adaptation, the pulse response during the odor background was divided by the pulse response in the absence of the odor background (e.g.,Fig. 4). The uncertainty in the adaptation factors from each cell was propagated from the SEMs of the parameters used to calculate the adaptation factor, using standard error propagation techniques (Taylor 1997; eq. (1)):

(2) $$\sigma_{\frac{\overset{¯}{x}}{\overset{¯}{y}}}=\frac{\overset{¯}{x}}{\overset{¯}{y}}\sqrt{{\left(\frac{\sigma_{\overset{¯}{x}}}{\overset{¯}{x}}\right)}^{2}+{\left(\frac{\sigma_{\overset{¯}{y}}}{\overset{¯}{y}}\right)}^{2}}$$

where $\overset{¯}{x}$ and $\overset{¯}{y}$ are the means of the sample distributions.

The average adaptation factors across cells were calculated as a weighted average; each cell's weight was inversely proportional to the uncertainty in that cell (eq. (1)):

(3) $$\left\langle \overset{¯}{x}\right\rangle =\frac{\sum (\frac{\overset{¯}{x}}{\sigma_{\overset{¯}{x}}^{2}})}{\sum (\frac{1}{\sigma_{\overset{¯}{x}}^{2}})}$$

This procedure biases the averages toward cells that have less uncertainty in their estimated adaptation factors. SEMs were calculated across the means in each cell.

**Figure 5:**
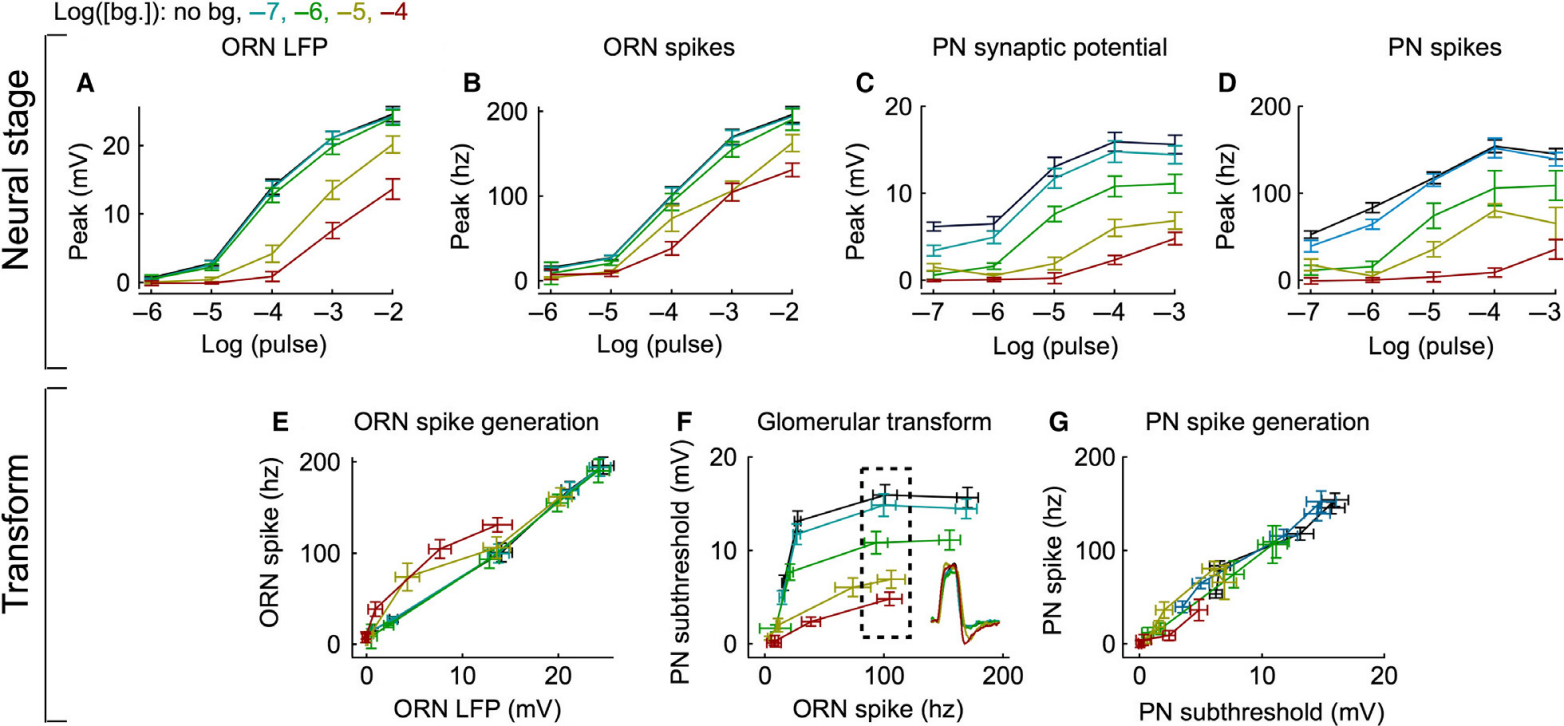
Adaptation of the pulse response across multiple odor pulse intensities. (A) The mean LFP peak pulse response curves (*N* = 5–9 cells, 3–21 trials per cell) for each background odor intensity calculated using the normalization procedure. Error bars indicate Standard error of the means (SEMs), propagated from paired data (Methods). (B) As in panel A, but using ORN spike rate data. (C) As in panel A, but using PN synaptic potential data (*N* = 5–9 cells, 3–10 trials per cell). (D) As in panel A, but using PN spike rate data. E‐G. Output of each stage plotted as a function of its input to assess the adaptation at a given stage. E. ORN LFP (Y‐values from panel A) plotted against ORN spikes (Y‐values from panel B), reflecting ORN spike generation F, ORN spikes (Y‐values from panel B) plotted against PN synaptic potential (Y‐values from panel C), reflecting the glomerular transform. Only responses to the same pulse value were plotted. The inset shows the average ORN spike pulse responses from the points within the dashed box. G, PN synaptic potential (Y‐values from panel C) plotted against PN spikes (Y‐values from panel D), reflecting PN spike generation.

To better illustrate adaptation in the pulse response, an average set of pulse response curves was calculated. The pulse response curves in the absence of background odor (see black curves in Fig. 5A‐D) were calculated by simply averaging across the pulse response in each cell. The pulse response curves in the presence of background odor (colored curves) were calculated by multiplying the pulse response curve in the absence of background odor by the average pulse adaptation factors measured at each odor stimulus condition. This procedure generates a set of pulse response curves that are similar to those created from simple averaging but are less distorted by cell‐to‐cell differences in response sensitivity (e.g., compare Fig. 4A green curve with points in 4B). The error bars in these curves were propagated from the uncertainty in the adaptation factors and the pulse responses in the absence of background odor (eq. (1)).

To examine the accuracy with which ORNs and PNs can detect background odors, the signal‐to‐noise ratio (SNR) was determined during the background odor response. The SNR was defined in each cell as the plateau of the background response divided by the standard deviation in the absence of an odor stimulus.

## Results

In *Drosophila,* most ORNs express a single member of a large family of olfactory receptors (for review, see Hallem et al. 2006). Each olfactory receptor is expressed by multiple ORNs; all ORNs expressing a given receptor project to a single glomerulus in the antennal lobe where they contact the second‐order neurons, PNs. Most PNs receive direct input from a single glomerulus. This study is focused on a single glomerulus, VM7, which receives projections from pb1A‐ORNs and VM7‐PNs (Fig. 2A, B). Responses of ORNs and PNs were measured using both a “private” odor and a “public” odor (see Methods for details; Fig. 1A). At low concentrations used in this study, 2‐butanone strongly activates pb1A and only weakly activates other ORN classes (Olsen et al. 2010), and hence is a private odor. Under these conditions, the pb1A ORN recordings reflected the dominant input to VM7 with minimal lateral input from other glomeruli. I later (Fig. 8) tested the generality of the findings using ethyl acetate at concentrations that strongly activates multiple ORN classes and evokes substantial lateral input from other glomeruli.

Figure 2 describes the experimental approach and illustrates the basic phenomena of interest. Four distinct signals were measured from the ORNs and the PNs: ORN local field potential (LFP), ORN spikes, PN synaptic potential, and PN spikes (Fig. 2A, B top). These measured signals reflect a preceding cascade of neural processes: Olfactory transduction, ORN spike generation, the glomerular transform, and PN spike generation (Fig. 2A, B, top). ORN activity was measured using single‐sensillum recordings from pb1 sensilla, which house the VM7 ORNs, on the fly's maxillary palp (Fig. 2A). ORN recordings yielded two signals: a slow local field potential (ORN LFP) and spikes. The ORN LFP was obtained by smoothing the voltage signal to remove the spikes. Previous work has shown that the ORN LFP reflects olfactory transduction (Nagel and Wilson 2011), although contributions from other conductances preceding spike generation have not been ruled out. The spiking responses of the ORNs depend both on the ORN LFP and spike‐generation mechanism in the ORN. Here, the term “spike generation” refers to the production of spikes from slow potentials (such as the LFP; Fig. 2A, B top).

PN activity was measured using whole‐cell patch‐clamp recordings from the VM7 PNs (Fig. 2B). As in the case of ORNs, both the filtered voltage and spike rates from PN voltage recordings were analyzed. Changes in the PNs filtered voltage are a measure of synaptic input into the PNs and are referred to in this paper as the “synaptic potential” (Fig. 2A, B top). The PN synaptic potential reflects the synaptic output of the ORNs, the intrinsic properties of the synapse, and the effect of local interneurons on both the ORNs and PNs. In this paper, I refer broadly to all mechanisms between the ORN spike output and PN synaptic potential as the “glomerular transform”. As in the ORNs, spike generation in the PNs can be affected through a variety of mechanism, including modification of intrinsic conductances (for review, see Lewis et al. 1986; Narusuye et al. 2003; Wark et al. 2007). Together, these four neural processes (olfactory transduction, ORN spike generation, the glomerular transform, and PN spike generation) constitute the fundamental components of early olfactory processing in the fly.

To examine adaptation, a brief (0.5 sec) odor pulse was delivered 10 sec after the onset of background odor stimulus (Fig. 2C top). ORN and PN spike rates were measured during stimulation with a private odor across a range of odor background and pulse intensities. Figure 2C shows the spike rate averaged across many ORNs and PNs during odor stimulation using a range of background intensities (0, 10^−7^, 10^−6^, 10^−5^, 10^−4^) and one pulse intensity (10^−4^).

**Figure 6:**
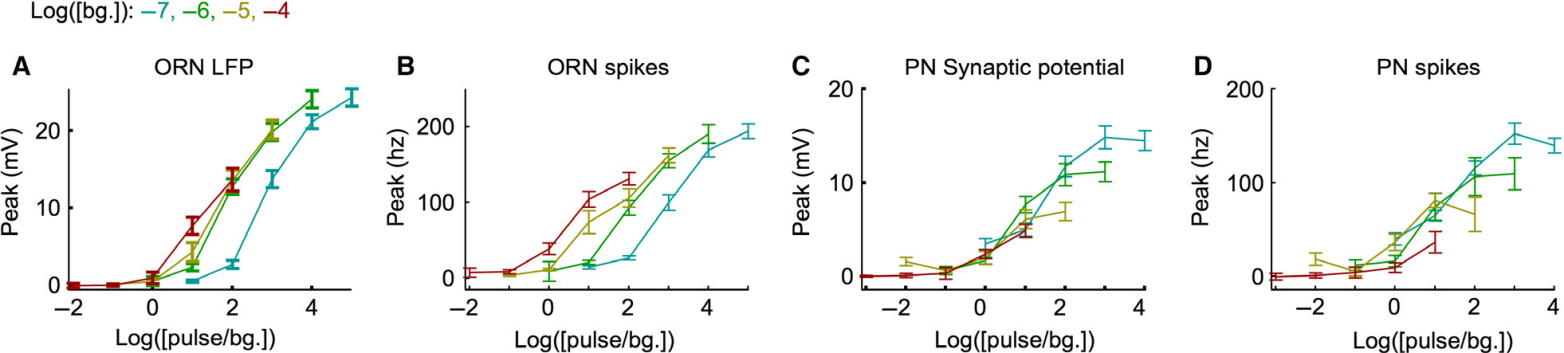
PNs encode contrast better than ORNs. (A) The mean LFP peak pulse response curves for each background odor intensity calculated as in Fig. 5A, but plotted against odor contrast (odor pulse intensity/odor background intensity), not pulse intensity. (B) As in panel A, but using ORN spike rate data. (C) As in panel A, but using PN synaptic potential data. (D) As in panel A, but using PN spike rate data.

**Figure 7:**
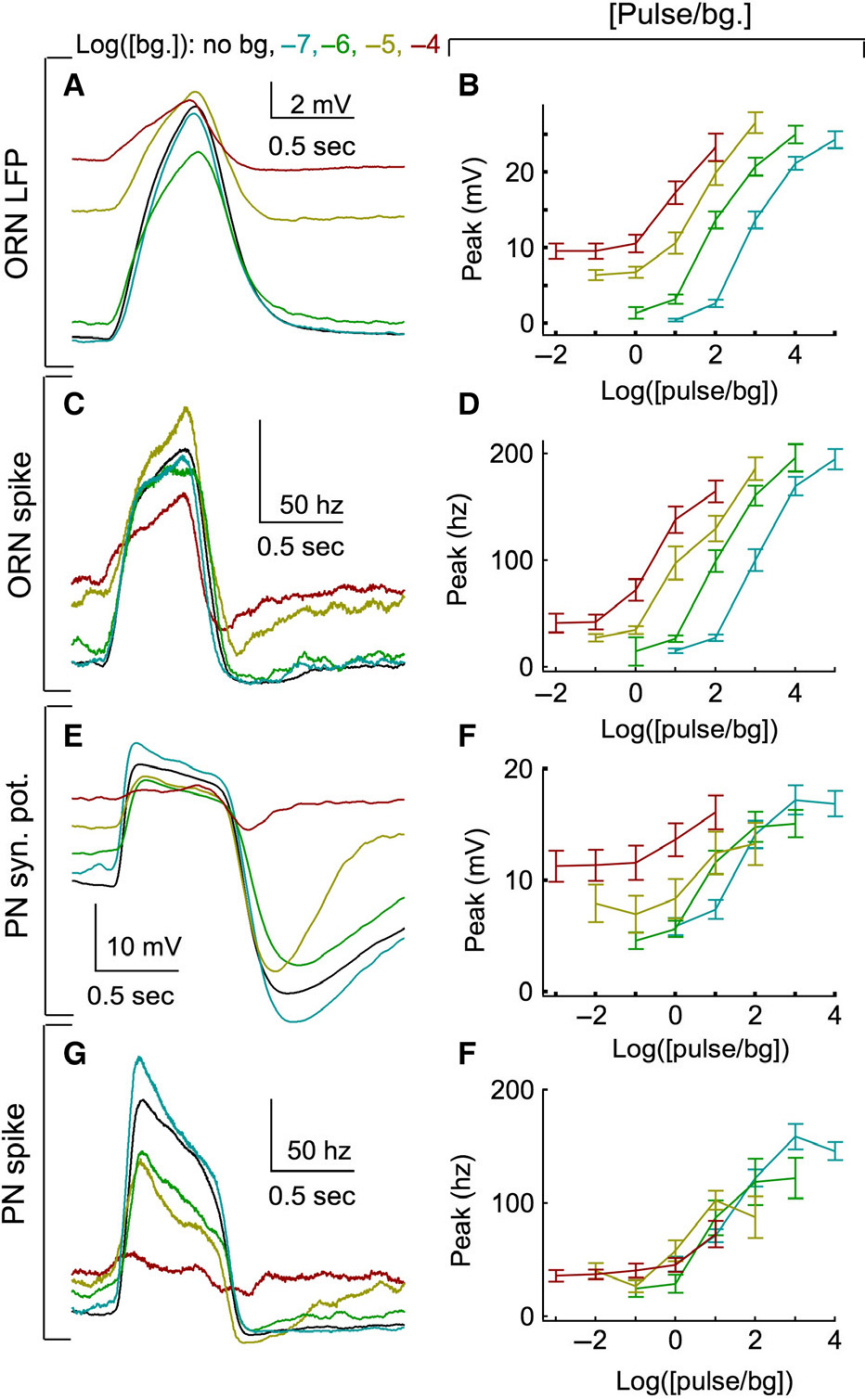
Full pulse response including background activity. **(**A) ORN LFP full pulse response averaged across trials (*N* = 4–11 trials) and cells (*N* = 5–6 cells) for the 10^−4^ odor pulse intensity. In this case, the response to the background odor was not subtracted from the pulse response as in Fig. 4A. B, The mean LFP peak full pulse response curves (*N* = 5–9 cells, 3–21 trials per cell) for each background odor intensity was calculated by adding the average background activity to the pulse response curves in Fig. 6A. Each response curve is plotted as a function of pulse odor contrast. C‐D, As in panels A‐B, but using ORN spike rate data. E‐F, As in panels A‐B, but using PN synaptic potential data. G‐H, As in panels A‐B, but using PN spike rate data.

Two forms of adaptation are evident in both ORN and PN spike responses (Fig. 2C). First, both cell types show transient responses that decay and plateau during the background odor (background adaptation). Second, both cell types show decreasing responsiveness to the pulse odor with increasing background odor intensity (pulse adaptation). Both background and pulse adaptation appear more prominent in the PN than the ORN spike rate (Fig. 2C). Below, I examine background (Fig. 3) and pulse (Fig. 4, 5, 6) adaptation in all four measured neural stages. Comparing the extent of adaptation at each stage reveals the origin of the observed adaptation. I also investigate the importance of both forms of adaptation in the ability of PNs to encode odor contrast (Fig. 7).

### Spike generation in both ORNs and PNs dominates background adaptation

In all four measured signals, the response to the background odor peaked and then decayed to a plateau (Fig. 3A,C). To compare the magnitude of background adaptation across multiple stages, the response was normalized to its peak value (Fig. 3A, C). The ratio of plateau to peak response at the four measured stages was calculated (Fig. 3B, D, E). A ratio of one indicates no background adaptation during the odor; a ratio of zero denotes complete background adaptation, indicating that the cell's activity level has returned to its spontaneous rate.

Except at the highest background odor concentration, there is only weak, inconsistent background adaptation in the ORN LFP (Fig. 3A, B, E). This suggests that olfactory transduction does not make substantial contributions to the background adaptation observed in later stages. Background adaptation increases from the ORN LFP to the ORN spike rate (Fig. 3A, B). Similarly, there is an increase in background adaptation between the PN synaptic potential and spike responses (Fig. 3C, D). Thus, the process of spike generation in both ORNs and PNs provides an important site for background adaptation.

Background adaptation is significantly greater in the PN spike rate than in the ORN spike rate (Fig. 3E; *P* < 0.05, unpaired t‐test). However, the magnitude of background adaptation did not consistently increase between the ORN spike response and the PN synaptic potential (Fig. 3E). In fact, on average, background adaptation was greater in the ORN spike response than the PN synaptic potential response at the highest background odor intensities (10^−5,−4^). This suggests that adaptation in the glomerular transform does not contribute significantly to background adaptation of the olfactory response. PN spike rates adapt more than ORN spike rates (Fig. 3E) because they benefit from adaptation in spike generation in both the ORNs and PNs.

### Olfactory transduction and the glomerular transform dominate pulse adaptation

Next, I examined pulse adaptation. The spike responses of both ORNs and PNs to a brief pulse of odor diminish in the presence of background odor (Fig. 2C). Do changes in spike generation underlie pulse adaptation, as they did background adaptation? To examine pulse adaptation independently of the background odor response, the background response plateau was subtracted from the measured full pulse response (this measure is referred to as the pulse response; see Methods). Figure 4 examines pulse adaptation at a single odor intensity (10^−4^) across a range of background odor intensities. At this pulse intensity, the mean pulse response decreases with increases in background odor at all four neural stages (Fig. 4A, D, G, J). The decrease in pulse response during odor background is evident in almost every cell tested (Fig. 4B, E, H, K).

Pulse adaptation factors were calculated by dividing the peak of the pulse response in the presence of the background odor by the peak in its absence (Fig. 4C, F, I, L). As with the background adaptation ratio (Fig. 3), a value of one indicates no pulse adaptation and a value of zero indicates complete pulse adaptation (i.e., no response to the pulse odor during a background odor). There are two important findings in these data. First, PNs adapts at lower background odor intensities than ORNs. ORN LFP and spike rates adapt significantly only at the two highest background odor intensities (10^−5,−4^; Fig. 4B, C, E, F). On the other hand, the PN synaptic potential and spike rate adapt at the two lowest background odor intensities (10^−7,−6^; Fig. 4H, I, K, L). Second, spike generation does not increase pulse adaptation as it did background adaptation. Spike rates in the PN and ORNs show pulse adaptation of similar magnitude or less than their preceding filtered voltage signals (ORN LFP and PN synaptic potential; Fig. 4F, L).

Figure 5 examines pulse adaptation across all measured pulse and background intensity combinations. To understand pulse adaptation within the context of the odor response, the measured pulse adaptation factors were used to calculate a set of response curves (Fig. 5A‐D; see Methods for details). Consistent with the earlier observation using a 10^−4^ pulse (Fig. 4), the ORN LFP response diminishes during the higher background odor intensities at all pulse concentrations measured (Fig. 5A). This result indicates that pulse adaptation occurs in the olfactory transduction cascade at the higher background odor intensities used in this study.

The decrease observed in the pulse response of the ORN LFP appears similar in magnitude to the decreases observed in the ORN spike rate (Fig. 5A, B). This suggests that ORN spike generation does not contribute much to pulse adaptation. To better compare pulse adaptation across neural stages, I assessed the “transforms” by plotting the output at that stage against its input from the preceding stage. If pulse adaptation occurs between two neural stages, then the transform will shift rightward with increasing background odor intensity (i.e., with increasing background odor, the same input will generate lower output). The transform between ORN LFP and spike rate reflects ORN spike generation. This transform was similar for the two lowest background odor intensities but showed leftward shifts for the two highest background odor intensities (Fig. 5E). These leftward shifts indicate a pulse sensitization (i.e., the same potential causes larger spike rates) not pulse adaptation. Because sensitization is observed in only a few comparable data points, I was unable to confidently assess its strength. Importantly, pulse adaptation is not observed within ORN spike generation at any pulse or background condition. Thus, the decrease in the ORN spike rate during background odors is inherited from pulse adaptation in olfactory transduction and ORN spike generation may underlie a small increase, not decrease, in the pulse response at high background odor intensities.

The pulse response in the PN synaptic potential is also diminished during the background odor (Fig. 5C). Qualitatively, the pulse adaptation observed in the PN synaptic potential was substantially more than that observed in the ORN spikes (Fig. 5B, C). The transform between ORN spikes and PN synaptic potential, defined as the glomerular transform, shows substantial reductions in gain with increased intensity of the background odor (i.e., the same ORN spike rate causes a smaller PN synaptic potential change; Fig. 5F). The ORN responses with similar peak spike rates had very similar response kinetics across odor backgrounds (Fig. 5F inset). This indicates that the diminished PN synaptic potential is caused by an adapting transformation, not changes in input kinetics. These results indicate that mechanisms operating in the glomerular transform are important for the pulse adaptation.

An important difference between pulse adaptation in the ORNs and PNs is that only PNs adapt at low background intensities (Fig. 5B, C). Lack of pulse adaptation in the ORNs at low background odor intensities could be a consequence of their low signal‐to‐noise ratio (SNR) at those intensities. Thus, integrating over multiple ORNs improves the SNR of the background response and allows the onset of adaptation at lower background intensities in the PNs. Indeed, at the lowest background odor intensity, the ORN spike rate SNR was only 0.06 ± 0.03; The PN spike rate SNR was 0.45 ± 0.08 and (see Methods).

Finally, I compared the pulse adaptation observed in the PN synaptic potential to that observed in the PN spike rate (Fig. 5C, D). The transform between PN synaptic potential and spike rate, reflecting PN spike generation, does not change with increased background (Fig. 5G). This indicates that PN spike generation contributes little to the observed pulse adaptation in the PN spike rate. Overall, adaptation in olfactory transduction (Fig. 5A) and the glomerular transform (Fig. 5F) is primarily responsible for the decrease in pulse response magnitude. Spike generation in the ORN (Fig. 5E) and PN (Fig. 5G) does not contribute greatly to the observed pulse adaptation.

Figures 4 and 5 plot pulse response (the background plateau response subtracted) as a function of pulse intensity. This is a standard way of examining pulse adaptation (see Laughlin et al. 1987 for discussion). However, it remains possible that the decrease observed in the pulse response is not adaptation to a background odor, but compression of the response along a fixed dose–response curve. This is not the case. As can be seen in Figure 2 (where the background was not subtracted), the pulse responses decrease with odor background, despite an increase in absolute odor concentration ([bg+pulse]). Indeed, curves plotting the response without the background subtracted against the total odor concentration ([pulse + bg]) show clear rightward shifts (data not shown). This indicates that the response is indeed an adaptive change, not a movement along a fixed dose–response curve. The implication of subtracting the background activity is further examined in Figure 7.

### Both pulse and background adaptation contribute to better contrast encoding in PNs

Neurons at many levels of the visual system follow Weber's law and encode pulse stimuli by contrast (pulse/background) and not the absolute intensity (for review, see Rieke and Rudd 2009). To examine how well ORNs and PNs encode odor contrast, I plotted the pulse response curves as a function of their contrast (Fig. 6A‐D). If pulse adaptation follows Weber's Law, the contrast–response functions at different background intensities will overlay each other (i.e., the response will depend only on the contrast not on the background intensity). The contrast–response curves for the ORN LFP appear to overlap at higher background odor intensities (>10^−7^; Fig. 6A), suggesting that olfactory transduction may approach Weber's law at higher background intensities. Much of the contrast–response curve overlap is lost in the ORN spike rate because of pulse sensitization during ORN spike generation (Fig. 6B). Owing to the strong pulse adaptation in the glomerular transform, the contrast–response curves overlap in the PN synaptic potential (Fig. 6C) and the PN spiking response (Fig. 6D). As a result, the PN synaptic potential and spike rate encode a much narrower range of contrasts at a given spike rate than observed in the ORN LFP or ORN spike response (Fig. 6C, D). Similarly, a given contrast is encoded by a much narrower range of spike rates in the PNs than in the ORNs (at log(1) contrast ORNs spike rate range ~90 ± 12 hz and PN spike rate range ~44 ± 20 hz). Thus, the PN pulse response encodes contrast with little ambiguity, regardless of pulse and background values, while the ORN pulse response does not.

Weber adaptation is traditionally assessed in the neuron's pulse response, as above, after subtracting the background activity. However, decoding of neural responses by higher order neurons may not involve a complete subtraction of the background spike rate. Indeed, neither the ORNs nor the PNs fully adapt out the background odor (Fig. 2, 3). Is contrast encoding maintained when the background activity is included?

Figure 7 examines the pulse response when background activity is not subtracted (herein referred to as the “full pulse response”). The full pulse response in the ORN LFP, ORN spike rate, and PN synaptic potential (Fig. 7A, C, E, F) adapts substantially less than the pulse response (Fig. 4A, D, G, J). This smaller adaptation reflects the two opposing effects of the background odor; the increase in neural activity caused by the background odor itself (see offsets Fig. 7A, C, E, G) compensates for the decrease in the response to subsequent pulses caused by the pulse adaptation (Fig. 4, 5). A consequence of this compensation is that the full pulse response–contrast curves of the PN synaptic potential are not independent of background intensity (Fig. 7F). Thus, the full pulse response of the PN synaptic potential does not narrowly encode odor contrast as when background activity was not considered (Fig. 6C). Only the PN spike response appears to faithfully encode odor contrast (Fig. 7H). This is because the PN spike response is affected by both pulse adaptation in the glomerular transform and background adaptation in spike generation. In other words, the background adaptation in PN spiking reduces the background response so that it can no longer compensate for the gain reduction in the pulse response. Thus, a combination of adaptive mechanisms is employed to maintain contrast encoding in the full pulse response of the PN output.

### Adaptation to a public odor is similar to adaptation to private odor

**Figure 8:**
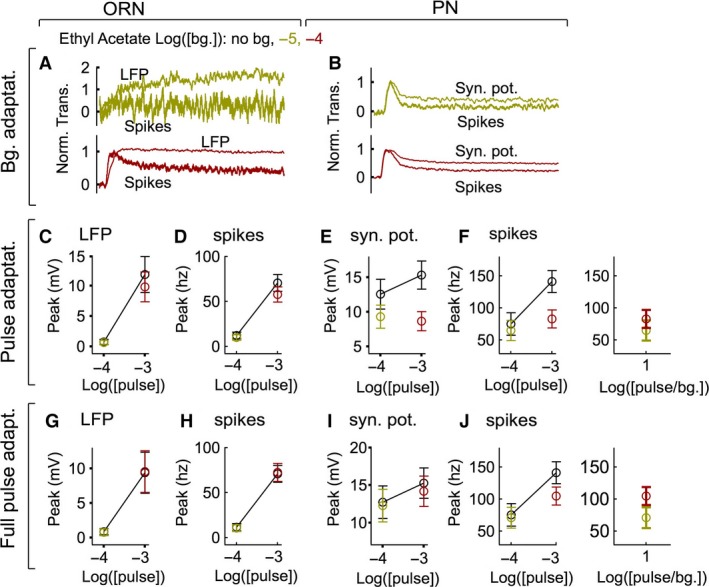
Adaptation during public odor (ethyl acetate) response. A, Adaptation to background odor in ORNs. As with the private odor (Fig. 3), ORN spikes adapt more than the LFP. B, PN spikes adapt more than the PN synaptic potential. C‐F. Pulse adaptation at the four sites studied here shows that pulse adaptation in the ORN (*N* = 5 cells, 10–20 trials per cell) is small, while that in PNs (*N* = 5 cells, 8–12 trials per cell) is more substantial. Adaptation at the level of synaptic potential contributes most to pulse adaptation. Similarity is evident between the PN spike response of the single contrast delivered. Right panel in F is same as left panel but response is plotted as function of pulse/bg. Background concentrations are color coded as in panel A. G‐J. As in panels C‐F, but using the full pulse response (without subtracting the background response) data. Adaptation is now most prominent in the PN spike rate.

Unlike a private odor, most odors strongly activate several types of ORNs. To examine if adaptation to a private odor is an adequate description for odor adaptation in general, I tested the previous findings with an odor that activates multiple ORN classes (Fig. 8). The same ORN (pb1A) and PN (VM7) classes were tested using ethyl acetate. Two pulse concentrations (10^−4^,10^−3^) and two background concentrations (10^−5^,10^−4^), were chosen to provide the same odor contrast (10^−4^/10^−5^, 10^−3^/10^−4^). At these concentrations, ethyl acetate is likely to activate multiple ORN classes strongly (Hallem and Carlson 2006; Olsen et al. 2010). Similar to the previous observations with 2‐butanone (Fig. 3), background adaptation during presentation of ethyl acetate is dominated by the spike generation step in ORNs (Fig. 8A) and PNs (Fig. 8B).

Pulse adaptation is also similar during public and private odors. A small pulse adaptation at the higher background odor intensity in the ORN LFP (Fig. 8C) is passed on to ORN spikes (Fig. 8D). On the other hand, there is a much larger pulse adaptation in both the PN synaptic potential (Fig. 8E) and PN spikes (Fig. 8F). Thus, similar to the observations using the private odor, much of this pulse adaptation occurs in the glomerular transform. Full pulse adaptation is also most prominent in the PN spike rate showing the same trend as the private odor (Fig. 8G‐J). The pulse response and full pulse response in PN spikes are very similar at the single tested contrast value (Fig. 8F, J right panels). These findings suggest that the key observations made using the public odor may generalize to other odors.

## Discussion

**Figure 9:**
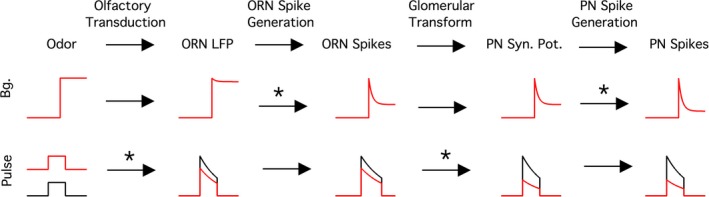
Summary of major sites of background and pulse adaptation. Simulated data of responses at each site with (red) and without (black) a background odor present. Major observed sites of adaptation are indicated with asterisks.

This study shows that exposure to an odor stimulus can trigger two distinct changes in the odor response of early olfactory neurons: (1) a slow decrease in spike rate, termed background adaptation, and (2) a decrease in sensitivity to a brief odor stimulus, termed pulse adaptation. Comparing these distinct adaptive changes across multiple stages in the ORNs and their cognate PNs, this study draws three important conclusions. First, PNs adapt at lower background odor intensities than ORNs. Second, background and pulse adaptation occur at different neural stages: background adaptation occurs largely during spike generation, while adaptation in olfactory transduction and the glomerular transform dominate pulse adaptation (Fig. 9). Third, PN spike rate encodes odor contrast with little ambiguity. Below, I discuss these findings in the context of other work on sensory adaptation.

### PNs adapt at lower stimulus levels than the ORNs

In this study, PNs but not ORNs adapt to the odor pulse at low background odor intensities (Fig. 4, 5). I attribute this difference to the higher signal‐to‐noise ratio (SNR) in the PNs. When averaged over long recording times, the ORNs do show an increase in mean firing rate to low background odor intensities, but the increase in the mean is obscured by their spontaneous activity, yielding a SNR <0.1 (i.e., the changes in the mean are smaller than ~45% of the noise fluctuations). Thus, an adaptive mechanism operating from the spike rate in the ORN would either often adapt in error or need to integrate over prohibitively long time periods. Because each VM7 PN receives input from ~40 independent ORNs, the PNs are able to increase their SNR enough to greatly improve the usefulness of an adaptive mechanism. The onset of adaptation at lower stimulus intensities in later neural stages has also been observed in the retina (Dunn et al. 2007). This suggests a general principle of sensory adaptation; convergence permits adaptation at lower stimulus intensities.

### Separate mechanisms underlie background and pulse adaptation

The results of this study indicate that background and pulse adaptation occur during distinct neural processes with different underlying mechanisms. Background adaptation was localized primarily within spike generation in both the ORNs and PNs. There is background adaptation observed in olfactory transduction at high background intensities (Fig. 3), consistent with previous reports in insects (e.g., Strausfeld and Kaissling 1986). However, this adaptation is relatively small when compared to that added by spike generation in the ORN and PN.

Adaptation in spike generation acted as a high pass filter and attenuated the response to the long‐lasting background odors without diminishing responses to brief odor pulses. In fact, spike generation even appears to increase the pulse response during a background odor (Fig. 4, 5). Spike generation in the frog olfactory receptors, however, appears to diminish responses to both background and pulse stimuli (Reisert and Matthews 1999). Thus, spike generation can play diverse roles in adaptation, even within homologous neurons under similar stimuli conditions.

Previous studies have observed a decrease in the steady‐state ORN spike rate during a constant odor stimulus (e.g., de Bruyne et al., 2001; Nagel and Wilson 2011) and a further decrease in the PN spike rate (Bhandawat et al. 2007). Indeed, spike generation was previously shown to play a role in ORN spike rate adaptation (Nagel and Wilson 2011). However, no previous study has compared decreases in the ORN spike rate with those in the PN synaptic potential. Thus, it was unclear if the difference observed between ORN and PN spike rate (Bhandawat et al. 2007) was caused by adaptive mechanisms within the ORN‐to‐PN synapse, PN spike generation, or both. By comparing odor responses across the ORN LFP, ORN spike rate, PN synaptic potential, and PN spike rate, I am able to draw two new conclusions: (1) the ORN‐to‐PN synapse does not contribute greatly to the reduction in steady‐state spike rate observed in the PN during an odor stimulus, and (2) spike generation in the PN plays a similar adaptive role to that observed in the ORN.

Different from background adaptation, pulse adaptation originated within olfactory transduction and the glomerular transform. Previous work observed a change in odor response gain within the ORN‐to‐PN synapse (Olsen et al. 2010). The odor pulse used in this previous work was targeted to a single ORN class, while a different background odor was presented simultaneously to strongly stimulate many other ORN classes. The background odor used in this study was presented prior to an odor pulse of the same odor and designed to minimize stimulation of other ORN classes. Thus, where previous research focused on the how lateral interactions help the olfactory system adapt to the molecular content of an odor stimulus, this study has focused on how a single olfactory channels adapt to the temporal components of an odor stimulus.

Pulse adaptation within the olfactory transduction cascade was significant, seemingly approaching Weber's law at higher background odor intensities (Fig. 6). However, some of the pulse adaptation in the ORN olfactory transduction cascade was negated by sensitization in spike generation. Spike sensitization was evident as leftward shifts in the ORN spike generation transformation (Fig. 5E), though its strength is difficult to assess with the current data set. The pulse adaptation observed in the ORN spike rate is similar to a previous study on adaptation in *Drosophila* ORNs (Martelli et al. 2013) but less than the pulse adaptation observed in frog ORNs (Reisert and Matthews 1999). The frog ORNs adapt more because, unlike *Drosophila* ORNs, pulse adaptation in both the transduction cascade and spike generation cooperate to strongly adapt the frog ORNs. Moreover, pulse adaptation in the olfactory transduction cascade observed in this study is less than that observed in frog ORNs (Reisert and Matthews 1999). This difference may result from differences in the transduction cascades. Olfactory transduction in vertebrates is mediated by G‐protein‐coupled receptors (for review, see Torre et al. 1995) but in *Drosophila* ORNs, transduction is more likely mediated by ionotropic receptors (for review, see Wilson 2013). However, adaptation in vertebrate photoreceptors, which also utilize a G‐protein‐coupled receptor cascade, follows Weber's law (Nakatani et al. 1991; Angueyra and Rieke 2013); thus, the reason for the difference in the frog ORNs remains unclear.

Pulse adaptation in the glomerular transform acts primarily as a low pass filter and adapts the odor response to the transient odor stimulus without significantly attenuating the response to the background odor. Synaptic depression at the ORN‐to‐PN synapse has been previously shown to decrease PN responsiveness during continued ORN spike trains (Kazama and Wilson 2008) and may be mediated by a depletion in presynaptic vesicles (Kazama and Wilson 2008) and by GABAergic presynaptic inhibition onto the ORNs (Olsen and Wilson 2008). Interestingly, a recent study suggests that inhibition also contributes to the lack of background adaptation observed within the glomerular transform (Nagel et al. 2014). Thus, presynaptic inhibition may serve the opposite role as spike generation, attenuating responses to transient stimuli without affecting responses to persistent stimuli.

These observations raise an important question: under what stimulus conditions might these adaptive mechanisms be correlated and under what stimulus conditions might they benefit from their separate underlying mechanisms? Natural visual (Rieke and Rudd 2009) and odor (Murlis et al. 2000) statistics suggest that the mean and variance of stimuli are often correlated. Adapting to both the background and transient stimuli during a persistent increase in background allows a sensory system to: (1) attenuate its response to persistent stimulus to preserve the neurons' response range and (2) reduce the systems response gain to better encode the increase in stimulus variance. These considerations may explain the correlated background and pulse adaptation observed in this study and studies in the visual system (Normann and Perlman 1979; Laughlin et al. 1987). An increase in stimulus background signals to the sensory system a probable increase in stimulus variance prompting a decrease in response gain. However, the mean and variance of sensory stimuli are unlikely to always be correlated. In this case, separate mechanisms to adapt to changes in the stimulus background independently from the transient changes, would allow the system to better encode the actual stimulus statistics (Fairhall et al. 2001; Mante et al. 2005). Odor statistics are greatly dependent on the environment (like wind, foliage density, and proximity to odor source and ground) and the odor mean and variance do not always increase proportionally (Murlis et al. 2000); thus, the olfactory system would benefit from having multiple sites that could adapt differently to distinct odor statistics. Indeed, previous work has shown multiple mechanisms of adaptation within the olfactory transduction cascade (Zufall and Leinders‐Zufall 2000) and the ORN (Strausfeld and Kaissling 1986; Kaissling et al. 1987).

### Odor encoding in the presence of background odor

These results have several important implications for odor encoding. In ORNs, a relatively small background adaptation results in a large steady‐state spike rate increase during a persistent odor stimuli. This increase in spike rate compensates for the limited pulse adaptation in the ORN spike rate. Consequentially, the full pulse response of the ORNs does not decrease proportionally with increasing background causing the ORN output to be a poor indicator of odor contrast.

PNs, on the other hand, encode odor contrast with little ambiguity. This result holds true if the steady‐state neural activity is either ignored (Fig. 6) or included (Fig. 7) during downstream decoding. To the best of my knowledge, this is the first study to report contrast encoding in the olfactory system. The PN response begins to deviate from zero when the pulse intensity matches the odor intensity (log contrast = 0; Fig. 6). It is important to note that such contrast–response curves are not necessarily fixed and could change under different stimulus conditions. As in the visual system (Ke et al., 2014), olfactory neurons may prove to have greater contrast sensitivity to stimuli that vary both above and below the background stimulus than only above, as examined in this study.

Regardless of contrast sensitivity, the ability of PNs to encode contrast suggests that olfactory behavior, like visual behavior, may follow Weber adaptation. If so, the odor concentration threshold needed to elicit a behavior will increase proportionally with the background odor concentration. A psychophysical study reported increased odor detection thresholds during a persistent odor background (Pryor et al. 1970) but did not directly compare these increases to Weber's law.

Most behavioral studies have focused on the loss of olfactory behavior after exposure to a persistent stimulus (Linster et al. 2007; Larkin et al. 2010; Murmu et al. 2011) and previous studies on adaptation in ORNs have reported that a background odor can effectively shut down ORN output (Reisert and Matthews 1999). Results in this study show that both ORNs and PNs continue to respond to both background and pulse odor stimuli. PNs, in particular, continue to generate responses significantly above their steady‐state firing rate to odor pulses even during the highest odor background (10^−4^). These results suggest that the adaptation observed in this study allows the olfactory system to continue to signal odor stimulation during changing odor environments. Thus, in some olfactory environments, shifts in detection threshold may better describe behavioral adaptation than total loss of odor detection. It is possible that adaptation downstream of the PNs undoes the contrast encoding observed in this study. Future studies should directly test for Weber adaptation in olfactory behavior.

## Disclosure

I declare no competing interests, financial, or otherwise.
